# Melatonin Supplementation for Cancer-Related Fatigue in Patients With Early Stage Breast Cancer Receiving Radiotherapy: A Double-Blind Placebo-Controlled Trial

**DOI:** 10.1093/oncolo/oyad250

**Published:** 2023-09-12

**Authors:** Nitai D Mukhopadhyay, Adam Khorasanchi, Sudeep Pandey, Srinidhi Nemani, Gwendolyn Parker, Xiaoyan Deng, Douglas W Arthur, Alfredo Urdaneta, Egidio Del Fabbro

**Affiliations:** Department of Biostatistics, Virginia Commonwealth University, Richmond, VA, USA; Department of Internal Medicine, Division of Hematology, Oncology, and Palliative Care, Virginia Commonwealth University, Richmond, VA, USA; Department of Internal Medicine, Division of Hematology, Oncology, and Palliative Care, Virginia Commonwealth University, Richmond, VA, USA; Department of Internal Medicine, Division of Hematology, Oncology, and Palliative Care, Virginia Commonwealth University, Richmond, VA, USA; Department of Radiation Oncology, Virginia Commonwealth University, Richmond, VA, USA; Department of Biostatistics, Virginia Commonwealth University, Richmond, VA, USA; Department of Radiation Oncology, Virginia Commonwealth University, Richmond, VA, USA; Department of Radiation Oncology, Virginia Commonwealth University, Richmond, VA, USA; Department of Internal Medicine, Division of Hematology, Oncology, and Palliative Care, Virginia Commonwealth University, Richmond, VA, USA; Department of Medicine, Medical College of Georgia, Augusta University, Augusta, GA, USA

**Keywords:** melatonin, radiation, fatigue, cancer

## Abstract

**Background:**

Fatigue is common in patients undergoing radiotherapy (RT) and can significantly impact quality of life. Melatonin, a safe inexpensive natural supplement, may improve symptoms and attenuate the side effects of RT. The purpose of this randomized double-blind placebo-controlled phase III trial was to assess the effects of melatonin for preventing fatigue and other symptoms in patients with breast cancer undergoing RT.

**Methods:**

Female early stage or Ductal carcinoma in situ patients with breast cancer ≥18 years of age with Eastern Cooperative Oncology Group (ECOG) performance status <3, hemoglobin ≥9 g/dL, planned for outpatient RT treatment with curative intent, were randomized 1:1 to melatonin 20 mg or placebo, orally, starting the night before RT initiation until 2 weeks post-RT. Randomization was stratified according to treatment duration (<3 weeks, ≥3 weeks) and prior chemotherapy. The primary endpoint was the Functional Assessment of Chronic Illness Therapy-Fatigue (FACIT-Fatigue scale), and secondary endpoints were FACIT-F subscales, Edmonton Symptom Assessment Scale (ESAS), and Patient-Reported Outcomes Measurement Information System (PROMIS) scores obtained at baseline, and 2 and 8 weeks post-RT. A 2-sided ANOVA *F*-test at a 4.5% significance level for the primary endpoint was used. Secondary analyses were reported using an *F*-test at a 5% significance level. The goal was to recruit approximately 140 patients with interim analysis planned mid-recruitment.

**Results:**

Eighty-five patients were screened for eligibility; 79 patients were randomized: 40 to melatonin and 39 to placebo; 78 patients were treated and included in the interim analysis at the mid-recruitment point. Baseline patient characteristics of age, race, and ECOG performance status were similar in both arms. The treatment effect was studied using a longitudinal mixed effects model with the effect of treatment over time (treatment × time) as the primary outcome parameter. The treatment × time for FACIT-Fatigue did not demonstrate statistical significance (*P*-value .83) in the melatonin group compared to placebo. In addition, secondary analyses of FACIT physical, social, emotional, and functional well-being scores did not demonstrate statistical significance (*P*-values of .35, .06, .62, and .71, respectively). Total PROMIS scores, collected as secondary outcome reported by patients, did not demonstrate statistically significant change over time either (*P*-value is .34). The other secondary scale, ESAS, was analyzed for each individual item and found to be nonsignificant, anxiety (*P* = .56), well-being (.82), drowsiness (.83), lack of appetite (.35), nausea (.79), pain (.50), shortness of breath (.77), sleep (.45), and tiredness (.56). Depression was the only item demonstrating statistical significance with a decrease of 0.01 unit in the placebo group, a change not considered clinically significant. Melatonin was well-tolerated with no grade 3 or 4 adverse events reported. The most common side effects were headache, somnolence, and abdominal pain. No patients died while participating in this study. Two patients died within a year of study completion from breast cancer recurrence. Sixteen patients withdrew prior to study completion for various reasons including adverse events, hospitalizations unrelated to study drug, RT discontinuation, and COVID-19 precautions.

**Conclusions:**

In this double-blind placebo-controlled phase III trial, melatonin did not prevent or significantly improve fatigue and other symptoms in patients with early stage breast cancer undergoing RT. The analysis, showing little evidence of an effect, at mid-recruitment, assured early termination of the trial.

## Teaser

Melatonin, a safe inexpensive natural supplement, often helps improve symptoms and attenuate the side effects of radiotherapy. This phase III trial assessed the effects of melatonin for preventing fatigue and other symptoms in patients with early stage breast cancer undergoing radiotherapy.

**Implications for Practice**

Use of melatonin has been widespread in managing symptoms of patients with cancer. As there is a lot of optimism about use of melatonin, very few blinded trials, which is the industry gold standard to establish clinical benefit of a new agent, have been reported. This study, although somewhat negative, reports a placebo-controlled double-blind study on the benefit of melatonin in managing fatigue; therefore, it is of immense importance for clinicians, as well as for patients going through cancer treatment.

## Introduction

Cancer-related fatigue (CRF) is common in patients undergoing treatment for cancer and can significantly impact quality of life (QOL).^1,2^ While the exact pathophysiology of CRF is not well defined, there are likely multiple contributing factors, including proinflammatory cytokines, hypothalamic-pituitary-adrenal (HPA) dysfunction, cachexia, and psychosocial factors such as chronic stress and depression.^3,4^

CRF is associated with radiotherapy (RT) for a number of different tumor types and is reported to be present in almost 90% of patients.^5^ Patients with early stage breast cancer receiving RT experience increased fatigue during treatment that can persist over 3 months following treatment completion.^6,7^ The prevalence, duration, and severity of fatigue in patients with breast cancer treated with RT depend on the type of RT administered, the irradiated volume, dose scheme, number of radiation fields, and the use of other treatment modalities.^8^ Heightened fatigue prior to treatment and an elevated interleukin-6 soluble receptor level are risk factors for increased fatigue during active RT.^9^ Factors such as stress, anxiety, depression, comorbidities, diarrhea, malnutrition, and anemia may further contribute to fatigue.^10^ Finally, an increased pretreatment fatigue level is a risk factor for persistent long-term fatigue following RT completion.^11^

Currently, there is no effective pharmacological therapy for CRF. Melatonin is an inexpensive, readily available, natural supplement shown to be radioprotective in animal models and safe in humans. Melatonin decreases the proinflammatory immune response, maintains circadian rhythm and sleep quality, and also prevents oxidative damage to mitochondria.^12,13^ Potential clinical benefits of melatonin supplementation in oncology include attenuating the side effects of RT and chemotherapy, improving symptoms, and prolonging survival.^14^ Systematic reviews and meta-analyses of melatonin therapy have reported improvement in tumor remission, 1-year survival, and fewer RT- and chemotherapy-related side effects.^15-17^ A meta-analysis of pooled data from 5 trials of patients receiving melatonin during concurrent chemoradiation reported a statistically decreased prevalence of fatigue compared to the control group.^16^ In addition, in 2 double-blind randomized controlled trials (RCT) in patients with breast cancer, melatonin was reported to significantly reduce depression in patients undergoing surgery and to provide a neuroprotective effect by counteracting the adverse effects of chemotherapy on cognitive function sleep quality and depressive symptoms.^18,19^ In contrast, RCTs of melatonin in patients with advanced cancers^20,21^ and for preventing recurrence in non-small cell lung cancer^22^ reported no improvement in QOL, fatigue, sleep, anxiety, or pain.

While melatonin failed to show benefits in patients with advanced cancer receiving palliative care,^20,21^ a strategy of earlier intervention and preventing radiation-induced side effects may be more effective at reducing symptom burden and improving QOL. The primary objective of this double-blind placebo-controlled phase III trial was to assess the effect of melatonin in the prevention of fatigue and other symptoms in patients with early stage breast cancer receiving RT.

## Methods

A double-blind placebo-controlled trial in patients with early stage breast cancer, randomized to 20-mg oral melatonin (Helsinn Chemicals, Biasca, Switzerland) or placebo starting the night before their first RT, continuing throughout RT and for an additional 2 weeks following completion of RT. Melatonin powder was prepared as a 2 mg/mL suspension in cherry flavored Ora-Plus/Ora-Sweet sugar-free vehicle. The suspension was packaged in a light-resistant amber bottle and stored at room temperature. The taste of the melatonin containing suspension was indistinguishable from the vehicle alone. Stratification in the randomization scheme balanced for prior chemotherapy and duration of RT (below 3 weeks or above). The primary objective was to determine the effect of melatonin on fatigue scores as measured by FACIT-Fatigue subscale from baseline to end of RT. The secondary outcomes were change in fatigue from baseline to 2 weeks, end of RT, and 8 weeks after completion of RT, as measured by PROMIS Fatigue scale and ESAS score. Also, the total and individual ESAS symptom scores between the 2 groups of patients were compared. In order to achieve 80% power at 5% level of significance, the final aim of the study was to recruit 71 patients in each of the placebo and melatonin groups. At mid-recruitment with 70 evaluable patients, an interim analysis was planned in a blinded manner, only to be shared with the Data Safety Monitoring Committee (DSMC). The final level of significance was adjusted using O’Brien Fleming alpha adjustment method. Upon DSMC recommendation, the study ended at interim analysis, and this report summarizes the efficacy and safety data of the cohort at the interim analysis.

### Selection of Patients

Ambulatory outpatient women ≥18 years of age with breast cancer (including Ductal carcinoma in situ) treated with RT for curative intent were approached for participation. Eligibility criteria included an Eastern Cooperative Oncology Group performance status <3 and hemoglobin ≥9 g/dL; postmenopausal or actively using birth control and not using melatonin prior to enrollment. All patients signed informed consent. Exclusion criteria included fatigue from other sources, such as hypothyroidism (TSH > 10 IU), hypercalcemia (calcium > 11 mg/dL), decompensated congestive heart failure, or chronic obstructive pulmonary disease requiring oxygen replacement. Patients with glomerular filtration rate (GFR) < 30 mL/minute, AST > 3 times the normal upper limit (ULN), ALT > 3 ULN, bilirubin > ULN, patients using systemic steroids, ginseng, ramelteon, or warfarin were excluded from the study. Following randomization, patients received daily melatonin or placebo beginning the night before their course of RT and an additional 2 weeks beyond the conclusion of their RT. For early stage breast cancer, patients received standard of care RT as determined by their physician (1 week of accelerated partial breast irradiation, 3-4 weeks of accelerated hypo fractionation RT or 6-8 weeks of standard RT). Patients were given a study diary to document compliance with the treatment regimen, and followed for 8 weeks after completion of RT or until death. Patients removed from the study due to unacceptable AEs were followed up until resolution of the AE.

### Statistical Analysis

**Figure 1.**
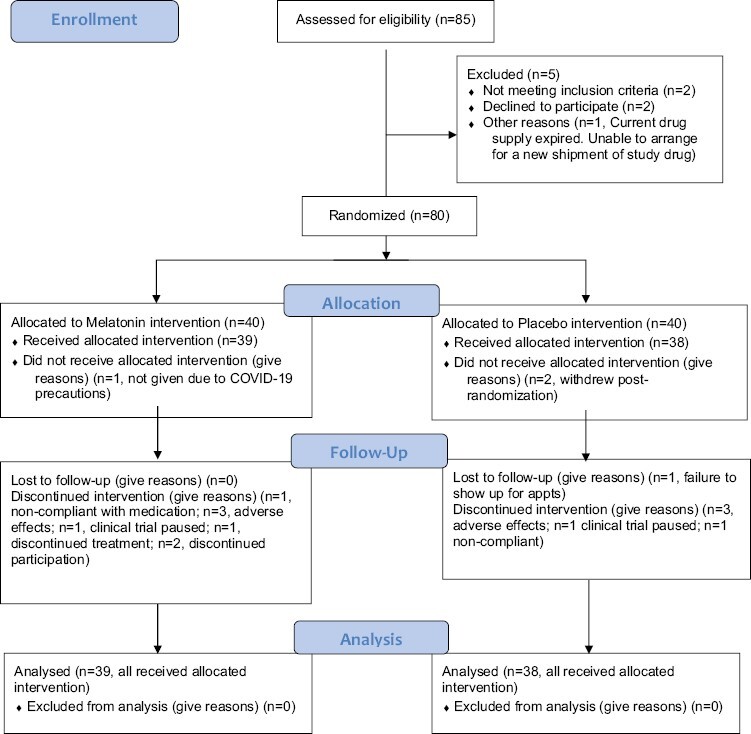
The CONSORT diagram displays the flow of patients through different stages of the study; 77 patients were included in the final analysis.

The study had an initial goal of 142 patients, with interim analysis at half recruitment. Upon recommendation from DSMC, the study concluded at interim analysis due to lack of efficacy. All the patients on study were followed up to completion, and the final database comprised all the data at the end of follow-up. The CONSORT diagram in Fig. 1 shows the flow of patients through different stages of the study and 78 patients with all the data considered for analysis. Table 1 provides the basic demographics of the cohort split into the treatment and placebo group. All the categorical attributes were compared using chi-square test, and all the continuous attributes were compared using 2 sample *t*-test. Analyses of primary and secondary outcomes were all analyzed using a longitudinal linear mixed effects model with the continuous score as the outcome and treatment, time and their interaction as the covariates with random effects introduced to model the dependence among the repeated measures from the same patient. All the statistical tests were performed at 5% level of significance using type III *F*-test. All analyses were performed utilizing R v4.1.2 and SAS v9.4 Statistical Software Package.

**Table 1.** Comparison of the demographics of patients enrolled in the melatonin and placebo treatment arm.

Category	Melatonin *N* = 40	Placebo *N* = 38	*P*-value
Age (year, median [range])	59.5 (35-73)	60.0 (38-78)	.391
Race (*n* [%])			
Black or African American	10 (25.0)	11 (29.0)	.7338
White	27 (67.5)	26 (68.4)	
Other	3 (7.5)	1 (2.6)	

The median age, race, and performance status of study patients were similar and demonstrated no statistically significant difference between the 2 groups.

## Results

Comparison of the basic demographics as reported in Table 1 shows no significant difference between the 2 groups. Both melatonin and placebo groups show no safety concerns. Table 2 shows the summary of all toxicities in each cohort. There was only one incidence of grade 3 insomnia that could be related to the study drug. None of the grade 4 or 5 toxicities were related to the study drug.

**Table 2.** Summary of all drug toxicities in each cohort.

Toxicity	Grade 3		Grade 4		Grade 5		Total	
	Melatonin	Placebo	Melatonin	Placebo	Melatonin	Placebo	Melatonin	Placebo
Vertigo	0	1	0	0	0	0	0	1
Fatigue	3	3	0	0	0	0	3	3
Pain	1	0	0	0	0	0	1	0
Breast infection	0	0	1	0	0	0	1	0
Dermatitis radiation	1	1	0	0	0	0	1	1
Hyponatremia	0	0	0	1	0	0	0	1
Myalgia	0	1	0	0	0	0	0	1
Dizziness	0	1	0	0	0	0	0	1
Headache	2	1	0	0	0	0	2	1
Nervous system disorders others, specify	0	1	0	0	0	0	0	1
Confusion	0	0	0	1	0	0	0	1
Depression	0	0	0	1	0	0	0	1
Insomnia	0	1	0	0	0	0	0	1
Acute kidney injury	0	1	0	0	0	0	0	1
Breast pain	1	0	0	0	0	0	1	0
Hot flashes	0	1	0	0	0	0	0	1
Hypertension	1	1	0	0	0	0	1	1

Nearly all patients experienced ≤grade 2 toxicities, with somnolence and headache as the most reported side effects. There is only one grade 3 insomnia that could be related to the study drug. None of the grade 4 or 5 toxicities are study drug related.

**Figure 2.**
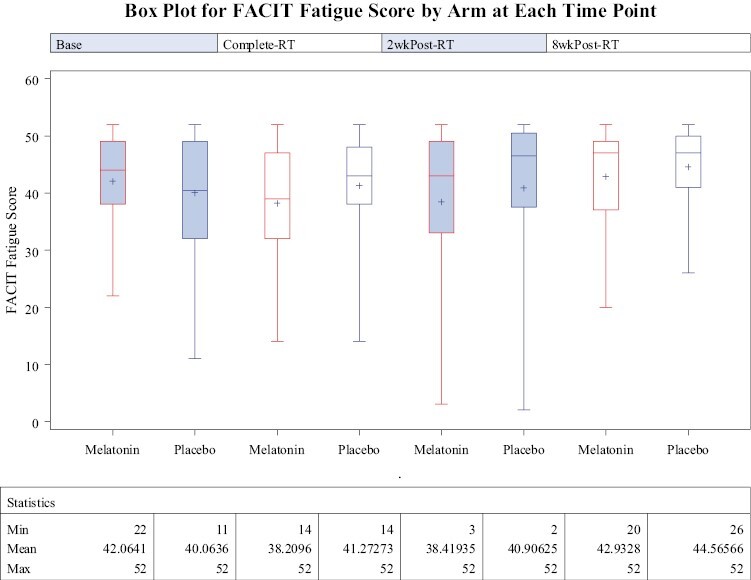
Box plot for FACIT fatigue scores of patients enrolled in the melatonin and placebo groups is shown at each time point (baseline, completion of RT, 2 weeks post-RT, and 8 weeks post-RT). Higher FACIT fatigue scores indicate less fatigue and, therefore, improved QOL. The treatment effect as measured by the treatment by time interaction was not found to be statistically significant (type III *P*-value is .8313).

Higher FACIT fatigue scores indicate less fatigue. Change from baseline in fatigue scores in each group is shown in Fig. 2. Treatment effect, as measured by the treatment by time interaction, has type III *P*-value of.8313, which is not statistically significant and confirms the pattern of little change demonstrated in Fig. 2. Secondary analysis of FACIT physical well-being score, social well-being, emotional well-being, and functional well-being shows a similar pattern of little change over time, and the same longitudinal model with the treatment by time interaction is not significant for time by treatment interaction (*P*-value .35 for physical well-being, .059 for social well-being, .626 for emotional well-being, and .708 for functional well-being).

**Figure 3.**
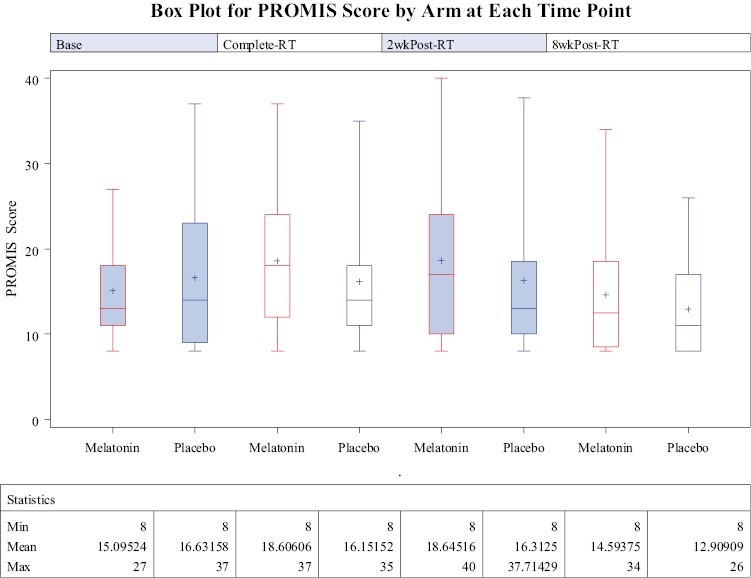
Box plot for PROMIS scores of patients enrolled in the melatonin and placebo groups is shown at each time point (baseline, completion of RT, 2 weeks post-RT, and 8 weeks post-RT). Higher scores indicate increased fatigue and therefore reduced QOL. The treatment effect as measured by the treatment by time interaction was not found to be statistically significant (*P*-value is .34).

For PROMIS fatigue short form, higher scores mean more fatigue. Total PROMIS scores over time is shown in Fig. 3, and the linear mixed effects model shows time by treatment effect to be not significant (*P*-value = .34).

ESAS score has 10 items for anxiety, well-being, depression, drowsiness, lack of appetite, nausea, pain, shortness of breath, sleep, and tiredness. Each individual score was analyzed with the same type of model and the treatment by time interaction was checked for statistical significance at 5% level. Depression was worse in melatonin group compared to placebo group with a difference of 0.01unit, which is not clinically meaningful, but statistically significant (*P*-value = .0389), while all other scores remained statistically not significant. As most of the findings indicate lack of any treatment effect, correction due to multiple tests was not performed.

## Discussion

Melatonin was not effective in preventing or decreasing fatigue in patients with early stage breast cancer undergoing RT. Other symptoms (except depression which worsened in the melatonin arm) also showed no difference compared with patients on placebo, and the trial was terminated at mid-recruitment, after interim analysis. This disappointing lack of benefit is in contrast with a recently published placebo-controlled RCT^23^ in 74 patients with breast cancer (stages I-III) receiving adjuvant chemotherapy, RT, and up to 26 weeks of melatonin. The Brief Fatigue Inventory score and severity of fatigue were significantly lower in the melatonin group 4 weeks after completion of adjuvant RT. While our trial measured fatigue scores at 2 and 6 weeks after completion of RT, the 2-week difference in interval is unlikely to cause the difference in outcomes; however, the longer duration of melatonin therapy and more advanced disease may contribute to improved symptom outcomes. Our cohort had lower symptom burden compared to the trial which included patients with locally advanced breast cancer. Our patients reported FACIT-Fatigue scores consistent with the general population^24^ and minimal severity (<3) ESAS fatigue scores at baseline. Patients with advanced metastatic disease may be refractory to the benefits of melatonin,^20,21^ but those with early stage may not derive significant benefit either because of relatively low symptom burden. Our trial has limitations. No serum levels of melatonin were performed. Serum levels may be important to confirm adherence to therapy and to evaluate the association between levels and clinical effect (great variability^25^ in inter-individual bioavailability has been documented in this regard). The use of a liquid vehicle requiring measurement by patients also increases the potential for dosing errors. Finally, the majority of study participants were older White adults, which may limit the generalizability of our findings. However, our trial has several strengths including the placebo-controlled design, the use of multiple outcome measures for fatigue (FACIT, ESAS, PROMIS), with results that were consistent across all measures, and the relatively low drop-out rate in comparison to patients with more advanced cancer.

## Conclusion

In patients with early stage breast cancer undergoing RT, melatonin did not decrease fatigue compared to placebo. Melatonin also demonstrated no benefit for other symptoms, leading to termination of the study at the mid-recruitment point.

## Data Availability

Aggregate data are available and uploaded to CT.gov. Patient level data are available upon request to the corresponding author upon institutional approval.
